# A Randomized Thorough QT Trial Using Concentration‐QT Analysis to Evaluate the Effects of Centanafadine on Cardiac Repolarization

**DOI:** 10.1002/cpdd.1545

**Published:** 2025-05-27

**Authors:** Osman S. Turkoglu, Xiaofeng Wang, Jennifer Repella‐Gordon, Susan E. Shoaf

**Affiliations:** ^1^ Otsuka Pharmaceutical Development & Commercialization, Inc. Rockville MD USA

**Keywords:** attention‐deficit/hyperactivity disorder, electrocardiograms, pharmacokinetics, QT prolongation, cardiac repolarization

## Abstract

Centanafadine is a norepinephrine/dopamine/serotonin reuptake inhibitor in development for treatment of attention‐deficit/hyperactivity disorder. This double‐blind, placebo‐ and moxifloxacin‐controlled, 3‐period crossover trial evaluated the effects of centanafadine (EB‐1020) and its metabolite (EB‐10601) on cardiac repolarization in 30 healthy adults (18‐65 years). Dosing sequences included centanafadine sustained‐release 800 mg (supratherapeutic) total daily dose, placebo, and moxifloxacin 400 mg. Electrocardiogram parameters and heart rate (HR) were assessed. The primary endpoint was placebo‐corrected change from baseline QTc (ΔΔQTc), analyzed using concentration‐QTc (C‐QTc) analysis. The C‐QTc slopes for centanafadine (−0.001 ms/[ng/mL]) and EB‐10601 (−0.0003 ms/[ng/mL]) were not statistically significant. Assay sensitivity was confirmed by the statistically significant C‐QTc slope for moxifloxacin (0.004 ms/[ng/mL]) and a 2‐sided 90% confidence interval lower bound >5 milliseconds. No change from baseline in QTcF or placebo‐corrected QTcF ≥10 milliseconds for centanafadine was observed at any postdose time point. No centanafadine‐treated participants had QTcF increases of >30 milliseconds and no relevant PR/QRS interval or HR increases were observed. The predicted ΔΔQTcF values of centanafadine, EB‐10601, and moxifloxacin at the geometric mean C_max_ were −2.72, −1.59, and 11.75 milliseconds, respectively. No serious treatment‐emergent adverse events or deaths were reported. Centanafadine was generally safe and well‐tolerated, with no clinically meaningful effect on cardiac repolarization.

Attention‐deficit/hyperactivity disorder (ADHD) is a neurodevelopmental disorder characterized by pervasive inattention and/or hyperactivity‐impulsivity.^1^ ADHD is often observed in childhood but can persist throughout an individual's lifetime and is associated with a multitude of mental health, behavioral, functional, and safety impairments.^2^, ^3^ As of 2020, it was estimated that ADHD affects more than 366 million adults worldwide.^4^ In the United States, it is estimated that over 8.7 million adults (4.4%) have been diagnosed with ADHD and approximately 6.5 million (10.5%) children and adolescents have current ADHD.^5^, ^6^

Centanafadine (EB‐1020) is a potential first‐in‐class norepinephrine/dopamine/serotonin reuptake inhibitor (NDSRI) in development for the treatment of ADHD.^7^, ^8^ In 4 phase 2 and 3 trials evaluating efficacy and safety in adults with ADHD, centanafadine, administered as a sustained‐release (SR) tablet twice daily (morning and 4‐6 hours later), was found to be efficacious, safe, and well tolerated at total daily doses (TDD) of 200 or 400 mg/day.^7^, ^8^

Pharmacokinetic (PK) studies indicate that, after SR tablet administration, peak centanafadine concentrations are reached within 1‐3 hours, with an elimination half‐life of approximately 4 hours. At a TDD of 200‐800 mg, linear pharmacokinetics are observed with twice‐daily dosing (q5h) and steady‐state concentrations are achieved by day 2, with minimal accumulation (<1.1‐fold). In vitro studies further demonstrated that centanafadine is metabolized via monoamine oxidase A, leading to the formation of EB‐10601. A mass balance trial confirmed that metabolism is the primary route of elimination, with EB‐10601 as the major metabolite (Figure 1).^9^ Notably, no clinically relevant changes in PKs were observed in individuals with moderate hepatic or severe renal impairment.^10^

**Figure 1:**
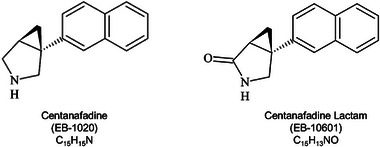
Structure of centanafadine (EB‐1020) and its metabolite EB‐10601.

In rat studies, centanafadine significantly increased brain concentrations of norepinephrine, dopamine, and serotonin in vivo.^11^ In a positron emission tomography trial in healthy male adults, centanafadine demonstrated occupancy of norepinephrine, dopamine, and serotonin reuptake transporters.^12^ Furthermore, centanafadine and its metabolite (EB‐10601) exhibited no in vitro functional activity at any other receptor, transporter, or ion channel at clinically relevant concentrations.

Given centanafadine's systemic bioavailability, it is essential to assess potential cardiac effects, particularly regarding QT interval prolongation.^13^, ^14^ Some non‐antiarrhythmic drugs are known to prolong the QT interval, which may lead to torsade de pointes, a polymorphic ventricular tachyarrhythmia that can progress to ventricular fibrillation and sudden death. To evaluate this risk, a phase 1 thorough QT (TQT) trial was conducted to assess the effects of centanafadine on cardiac repolarization, specifically by measuring the QTc interval (QT corrected for heart rate [HR]) in healthy adults.

## Methods

The trial was conducted at ICON, Salt Lake City, UT, USA. The institutional review board/independent ethics committee for the trial site (Advarra, Columbia, MD, USA) approved the protocol before enrollment. This trial was conducted in accordance with Good Clinical Practice guidelines and ethical principles derived from the Declaration of Helsinki. All trial participants provided written informed consent before participating.

### Trial Design

This phase 1, single‐site, double‐blind, randomized, placebo‐ and active‐controlled (moxifloxacin), 3‐period crossover TQT trial, conducted from March 2019 to May 2019, evaluated the effect of centanafadine exposures on cardiac repolarization in healthy male and female adults. The 42‐day trial consisted of a screening phase (day −28 to day −2), check‐in (day −2), an in‐clinic baseline day (day −1), a 9‐day in‐clinic treatment period, and a safety follow‐up telephone call 7 (+2) days after the last dose of study medication (Figure S1).

Treatment assignments were based on a computer‐generated randomization code provided by the Otsuka Pharmaceutical Development & Commercialization, Inc., Biometrics Department. Only sponsor personnel charged with generating and maintaining randomization files and the unblinded pharmacist who prepared the treatments at the trial site had access to the randomization files. The trial evaluated the QT interval using a positive control, moxifloxacin,^14^ to ensure that small changes in the QT interval could be detected.

### Participants

It was expected that 30 healthy adults would be enrolled. Men and women aged 18‐65 years were eligible if they had a body mass index of 19.0‐32.0 kg/m^2^, were in good health as determined by medical history and physical examination, electrocardiography (ECG), and serum/urine biochemistry, hematology, and serology test results, were able to provide written informed consent, and agreed to use 2 different approved methods of birth control or practice abstinence during the trial and for 30 days thereafter.

Potential participants were excluded if they had a clinically relevant abnormality in their past medical history or findings at screening that may place them at risk (eg, myocardial infarction, cardiac surgery revascularization, angina, cerebrovascular accident or stroke, use of a pacemaker) or that could interfere with outcome variables including absorption, distribution, metabolism, and excretion of drug, a history of unexplained syncope, a personal or family history of sudden death or long QT syndrome, or abnormal ECG findings at screening or check‐in (QTc interval using the Fridericia correction formula [QTcF] >450 milliseconds for male participants, QTcF >470 milliseconds for female participants, QRS interval >120 milliseconds, PR interval >200 milliseconds, abnormal U waves, or other minor ST‐ or T‐wave changes) that were considered clinically significant. For additional detailed exclusion criteria, see the Supplemental Information.

### Treatments

Centanafadine SR tablets, moxifloxacin (400‐mg tablets manufactured by Alvogen), or matching centanafadine placebo were administered orally in covered cups in a double‐blind manner. Placebo tablets were added to the cups that contained the positive control, moxifloxacin, so that the total number of tablets was the same across treatments. The maximum dose of centanafadine SR evaluated in phase 3 randomized, placebo‐controlled clinical trials in adults was 400 mg TDD.^7^ Based on the results of a previously conducted drug–drug interaction trial and on safety data obtained from previous phase 1 trials, assessment of QT prolongation risk with centanafadine SR was conducted at the supratherapeutic dose of 800 mg TDD.

Centanafadine SR 800 mg TDD (treatment A) was administered as 400 mg twice daily (4 × 100‐mg tablets in the morning and 4 × 100‐mg tablets 5 hours later), centanafadine placebo tablets (treatment B) were given twice daily (in the morning and 5 hours later), and moxifloxacin 400 mg (1 × 400‐mg tablet) was administered with centanafadine placebo tablets in the morning followed by centanafadine placebo tablets 5 hours later (treatment C) in a crossover design on days 1, 4, and 7. Morning doses were administered while the participant was fasting. Participants were randomized to 1 of 6 treatment sequences (ABC, ACB, BAC, BCA, CAB, or CBA) in a full‐block William's square design, which is a balanced crossover design when there are more than 2 treatments in a trial.^15^ Two randomization blocks (6 participants per block) were used for 12 women, 2 randomization blocks were used for 12 men, and another randomization block was used for the remaining 6 participants.

### ECG Assessments

All participants who received a dose of centanafadine SR or moxifloxacin and had QTc measurements at baseline and after dosing were included in the analyses. On day −1, continuous digital ECG monitoring was initiated and continued so that 12‐lead ECGs could be extracted from the continuous recordings at −0.75, −0.50, and −0.25 hours prior to planned time 0, and at 1, 2, 3, 4, 5, 6, 7, 8, 10, 12, and 16 hours thereafter. For each treatment period (days 1, 4, and 7), continuous digital 12‐lead ECG monitoring was initiated at least 45 minutes prior to dosing and continued for at least 24 hours after the morning dose so that 12‐lead ECGs could be extracted from the continuous recordings at predose (ECG readings at −0.75, −0.50, and −0.25 hours prior to the treatment) and at 1, 2, 3, 4, 5, 6, 7, 8, 10, 12, 16, and 24 hours after the first dose of treatment in each period. Participants were maintained in a supine or semi‐recumbent quiet position for 15 minutes before the ECG extraction period. Three ECG replicates were extracted in the next 5 minutes, with at least 1 minute between each extracted ECG, and were averaged for use in the analyses. Criteria for potentially clinically significant ECG measures were QTcF >450 milliseconds for male participants, >470 milliseconds for female participants, QRS interval >120 milliseconds, and PR interval >200 milliseconds. Criteria for potentially clinically significant vital signs included HR <50 beats per minute or >90 beats per minute.

### Pharmacokinetic Analysis

Plasma samples for PK analyses were collected at the same time points for 24 hours after the morning dose in each period, as was done for the ECG collection. For periods 1 and 2, additional plasma samples were collected at 30, 36, 48, and 60 hours after the morning dose, while in period 3, additional plasma samples were collected at 30, 36, and 48 hours after the morning dose.

The PK analysis data set included participants who received a dose of centanafadine SR or moxifloxacin and had evaluable plasma concentration data. Plasma concentrations were analyzed for centanafadine and its metabolite, and moxifloxacin using a noncompartmental analysis with actual times postdose. Concentrations below the limit of quantification after the last measurable concentration were excluded from the analysis. Values for maximum plasma concentration (C_max_) and time to maximum plasma concentration (t_max_) were determined directly from the observed data. The terminal phase elimination rate constant (λ_z_) was estimated by a log‐linear regression of at least 3 non‐0 concentrations; regressions with r^2^ adjusted values <0.8 were not reported. Values for area under the concentration‐time curve from time 0 to infinity were estimated using the linear trapezoidal rule and last observed measurable plasma concentration. Descriptive statistics and plasma PK parameters were determined using SAS Version 9.4 (TS1M4).

The ECG assessments (QT/corrected QT interval [QTc]) included all participants who received at least 1 dose of centanafadine or moxifloxacin and had measurements at baseline and on treatment, with at least 1 postdose time point with a valid change from baseline in the QTcF (ΔQTcF) value. The PK and ECG assessment data set included all participants who had baseline and postdose QT assessments, with at least 1 plasma concentration available at the same postdose time point.

### Bioanalytical Analysis

Blood samples for measurement of centanafadine and the EB‐10601 metabolite plasma concentrations were collected in 4‐mL vacutainer tubes containing dipotassium ethylenediaminetetraacetic acid (K_2_EDTA) as an anticoagulant. Centanafadine, its metabolite, and spiked internal standards (^13^C‐centanafadine and ^13^C‐EB‐10601) were extracted by protein precipitation with methanol from 100 µL of plasma containing K_2_EDTA. The analytes and internal standards were chromatographically separated using reversed‐phase high‐performance liquid chromatography and detected by tandem mass spectrometry (MS) in positive ion mode using multiple reaction monitoring. Samples were injected onto a Waters XBridge C18 3.5‐µm, 2.1 × 50 mm analytical column, and analytes were eluted using a formic acid mobile phase A and an acetonitrile mobile phase B. Monitored MS transitions were centanafadine, m/z 210.12→169.20; EB‐10601, m/z 224.11→141.10; ^13^C‐centanafadine, m/z 216.15→175.20; ^13^C‐EB‐10601, m/z 230.13→147.30. Calibration standards were used to extrapolate the concentrations of the analytes by weighted (1/x^2^) linear regression of peak area ratios of analyte‐to‐internal standard over a range of 5.00‐1500 ng/mL. Quality control samples at 3 concentrations, as well as at the lower limit of quantitation (5.00 ng/mL), were assayed to monitor analytical performance. Acceptable accuracy, precision, linearity, and specificity were demonstrated. Assay performance data met the pre‐specified acceptance criteria of interassay precision ≤15% and interassay accuracy of ±15% of nominal. Stability experiments were evaluated, and data showed that the analytes were stable under the experimental conditions of the assay.

Blood samples for measurement of moxifloxacin plasma concentrations were collected in 4‐mL vacutainer tubes containing K_2_EDTA. Moxifloxacin‐d_4_ was used as the internal standard. For moxifloxacin, samples were injected on a Phenomenex Gemini NX C18 3‐µm, 2.0 × 50 mm analytical column, and analytes were eluted using an ammonium formate, pH 3.0 mobile phase A and acetonitrile mobile phase B. Monitored MS transitions were moxifloxacin, m/z 402.18→384.17; moxifloxacin‐d_4_, m/z 402.21→388.20. Quality control samples at 3 concentrations, as well as the lower limit of quantification, were assayed to monitor analytical performance. The lower limit of quantification for moxifloxacin was 10.0 ng/mL. Acceptable accuracy, precision, linearity, and specificity were demonstrated. Assay performance data met the pre‐specified criteria of interassay precision ≤15% and interassay accuracy ±15% of nominal. Stability experiments were evaluated, and data showed that the analytes were stable under the experimental conditions of the assay.

Validation of the plasma assays for centanafadine, of the EB‐10601 metabolite, and of moxifloxacin was performed at Altasciences (Laval, Quebec, Canada).

### Safety

All participants who received a dose of centanafadine were included in the safety analysis. Safety endpoints included adverse events, clinical laboratory test results (hematology, serum chemistry, and urinalysis), vital sign measurements, 12‐lead ECG, and the Columbia‐Suicide Severity Rating Scale (C‐SSRS). Any vital sign or ECG abnormalities considered by the investigator to be clinically significant were recorded as adverse events (AEs) on an AE electronic case report form.

### Statistical Analysis

Based on experience from the Innovation and Quality Cardiac Safety Research Consortium study^16^ and on simulations to evaluate the power of small trials,^17^ a sample size of 24 participants would provide more than 96.5% power to exclude more than a 10 milliseconds QTc effect with centanafadine at clinically relevant plasma levels, as demonstrated by the upper bound of the 2‐sided 90% confidence interval (CI) of the model predicted QT effect falling below 10 milliseconds. The calculation assumed a small underlying effect of centanafadine of 3 milliseconds and a standard deviation of the change from baseline QTcF (ΔQTcF) of 7 milliseconds. Assuming a dropout rate of about 20%, 30 participants randomized to 6 sequences of the trial would ensure 24 completers, providing at least 80% power to demonstrate assay sensitivity using exposure‐response analysis.

The primary endpoint was the placebo‐corrected change from baseline QTc (ie, ΔΔQTcF). Secondary endpoints included time‐matched change from baseline in QTcF (ΔQTcF) and changes from baseline in HR (ΔHR) and in PR and QRS intervals. The relationship between ΔΔQTcF and plasma concentrations of centanafadine and the EB‐10601 metabolite was evaluated using a linear mixed‐effects modeling approach.

$$\begin{array}{ccc} \Delta \Delta \mathrm{QT}C_{\mathrm{ij}} & = & \alpha +S_{i}+\left(\beta +d_{i}\right)\times \mathrm{Cct}\\ & & n_{\mathrm{ij}}+\left(\gamma +h_{i}\right)\times \mathrm{Cla}c_{\mathrm{ij}}+\varepsilon_{\mathrm{ij}} \end{array}$$

In the above model, Cctn_ij_ and Clac_ij_ are plasma concentrations of centanafadine and the EB‐10601 metabolite for the ith participant at the jth time point, respectively. The parameter α is the population mean intercept, and β and γ are the population mean slopes of centanafadine and its metabolite, respectively. The parameter s_i_ is the random effect of participant i on the intercept, and d_i_ and h_i_ are the random effects of participant i on the slopes. The random effects *S*_i_, d_i_ and h_i_ are assumed to be independent and identically distributed (iid) trivariate normal TVN(0, Σ), where Σ is a 3 × 3 covariance matrix. The error term ɛ_ij_ is assumed to be iid normal N(0, σ^2^).

Key assumptions for the concentration corrected QT (C‐QTc) analysis were that (1) centanafadine would have no effect on HR, (2) the corrected QT (QTc) interval would be independent of HR, (3) there would be no time delay (hysteresis) between drug concentration and ΔΔQTcF, and (4) there would be a linear concentration‐QTc relationship.

Assay sensitivity was determined by a similar exposure‐response analysis of 400 mg oral moxifloxacin data. The by‐time‐point analyses for ΔHR and ΔQTc were based on a linear mixed‐effects model analysis of covariance with ΔHR and ΔQTc, respectively, as the dependent variables. Period, sequence, time (categorical), treatment (centanafadine, moxifloxacin, or placebo), and time‐by‐treatment interaction were factors, baseline HR (for ΔHR) and baseline QTc were covariates; participants were included as a random effect for the intercept. Least squares mean and 2‐sided 90% CIs were calculated for the comparison of centanafadine and the EB‐10601 metabolite versus placebo at each postbaseline time point, separately, using the ECG assessment data set. Safety endpoints were reported descriptively.

## Results

### Participant Characteristics

A total of 30 participants were randomized to 1 of 6 treatment sequences (5 participants per sequence). In total, 29 participants received centanafadine SR, 30 received moxifloxacin, and 30 received placebo. Participants were mostly male (56.7%) and White (86.7%), with a mean age of 37.6 years (Table S1). Of the randomized participants, 27 (90%) completed the trial and 3 (10%) withdrew for personal reasons.

### Centanafadine Pharmacokinetics

**Figure 2:**
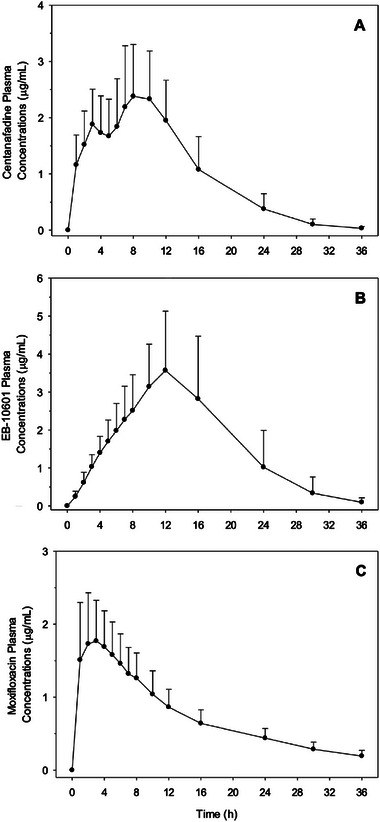
Mean (SD) plasma concentrations of centanafadine (A) and EB‐10601 (B) after a 800‐mg TDD of centanafadine SR and of moxifloxacin (C) after a 400‐mg single dose of moxifloxacin to healthy adults, n = 29 for centanafadine and EB‐10601 and n = 30 for moxifloxacin; lower limit of quantification in plasma was 5.00 ng/mL for centanafadine and the EB‐10601 metabolite, and 10.0 ng/mL for moxifloxacin. SD, standard deviation; SR, sustained release; TDD, total daily dose.

When assessed over the 36‐hour period following administration of centanafadine SR 800 mg TDD, median plasma concentration of centanafadine (Figure 2A) peaked at 8 hours (2.48 µg/mL), while EB‐10601 concentration (Figure 2B) peaked at 12 hours (3.22 µg/mL). Median plasma concentration of moxifloxacin (Figure 2C) following administration of moxifloxacin 400 mg single dose peaked at 2 hours (1.59 µg/mL). Pharmacokinetic parameters for centanafadine and EB‐10601 following an 800‐mg TDD of centanafadine SR, and of moxifloxacin following 400 mg single dose of moxifloxacin are presented in Table 1.

**Table 1:** Pharmacokinetic Parameters for Centanafadine and EB‐10601 in Plasma Following Administration of an 800‐mg TDD of Centanafadine SR and for Moxifloxacin in Plasma Following Administration of a 400‐mg Single Dose of Moxifloxacin to Healthy Adults

Parameter^a^	Centanafadine (n = 29)	EB‐10601 metabolite (n = 29)	Moxifloxacin (n = 30)
C_max_ (µg/mL)	3.00 (0.936)	3.75 (1.50)	2.04 (0.61)
t_max_ (hour)	8.00 (3.00‐16.00)	12.00 (6.00‐16.03)	2.00 (1.00‐4.92)
AUC_∞_ (µg•h/mL)	36.0 (10.3)^b^	57.1 (27.8)	31.1 (8.8)^b^
t_½,z_ (hour)	3.5 (1.7)^b^	3.6 (1.2)	13.2 (1.8)^b^

AUC_∞_, area under the plasma‐concentration curve from time 0 to infinity; C_max_, maximum plasma concentration; SR, sustained release; t_½,z_, terminal elimination half‐life; TDD, total daily dose; t_max_, time to maximum plasma concentration.

a Values are mean (standard deviation) except for t_max_, which is reported as median (range).

b n = 28

### ECG Profile

**Figure 3:**
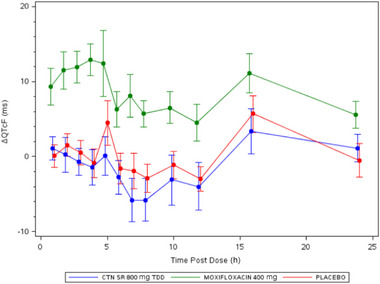
LS mean difference (90% CI) for time‐matched ΔQTcF by treatment, ΔQTcF, change from baseline in QTcF; CI, confidence interval; CTN SR 800 mg TDD, centanafadine sustained release tablet 800 mg total daily dose; LS, least squares.

Least squares mean ΔQTcF values at each time point over 24 hours following each treatment are presented in Figure 3. Overall, the mean ΔQTcF for centanafadine ranged from −5.8 to 3.4 milliseconds and was lower than with moxifloxacin, which ranged from 4.5 to 12.9 milliseconds. The largest mean ΔQTcF for centanafadine occurred at hour 16 postdose and the smallest occurred at hours 7 and 8 postdose. The largest and smallest mean ΔQTcF for moxifloxacin occurred at hours 4 and 12 postdose, respectively. The mean ΔΔPR for centanafadine ranged from −9.7 to −5.0 milliseconds, with the largest occurring at hour 6 and smallest at hour 24; ranges for moxifloxacin were from −3.9 to 2.0 milliseconds, with the largest occurring at hour 12 and smallest at hour 24. The largest mean ΔΔQRS for centanafadine was 0.9 milliseconds at hour 3, while the smallest was 0.3 millisesonds at hour 6; for moxifloxacin the largest ΔΔQRS was −0.7 milliseconds at hours 4 and 5 and the smallest was 0.4 milliseconds at hour 3.

No participant taking centanafadine had a QTcF increase of >30 milliseconds, a PR increase of >25% and >200 milliseconds, a QRS increase >25% and >120 milliseconds, or an HR increase of >25% and >100 beats per minute (bpm). Four participants had a >25% decrease in HR that was <50 bpm. One participant on centanafadine had abnormal T‐wave morphology; no abnormal U‐wave morphology was observed.

### Concentration‐QTc Analysis

#### Evaluation of Model Assumptions

Results obtained after administration of centanafadine SR 800 mg TDD confirmed that all 4 model assumptions were met. The largest placebo‐corrected mean ΔHR (ie, ΔΔHR) was not greater than 10 bpm, signifying no meaningful effect of centanafadine or EB‐10601 plasma concentrations on HR (Figure S2). Because no apparent relationship existed between QTcF and the corresponding RR interval values at predose time points (−0.75, −0.50, and −0.25 hours) or while on treatment (Figure S3), the QTcF interval was considered independent of HR. Given that hysteresis curves for mean ΔΔQTcF versus centanafadine and the EB‐10601 metabolite concentrations did not contain a counterclockwise loop (Figure S4), no time delay between ΔΔQTcF and centanafadine concentrations was present. Lastly, the overlap of the linear regression line, quantiles, and locally weighted scatterplot smoothing line in the time‐matched scatterplot of ΔΔQTcF and centanafadine concentration (Figure S5) indicated that the concentration‐QTc relationship was linear.

#### Linear Mixed‐Effects Model

**Figure 4:**
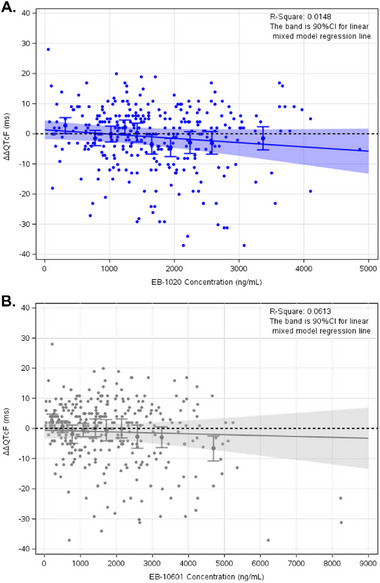
Observed data and quantiles overlaid with linear mixed‐effects model predicted ΔΔQTcF for centanafadine (A) and EB‐10601 metabolite (B). Solid lines are linear mixed regression lines and shaded areas are the 90% CI. Solid circles denote individual data. Quantiles denote mean concentration and mean ΔΔQTcF (90% CI) for each bin. ΔΔQTcF, placebo‐corrected change from baseline in QTcF (ie, QT interval corrected using the Fridericia correction formula); CI, confidence interval.

The relationships between ΔΔQTcF and centanafadine concentration and between ΔΔQTcF and the EB‐10601 metabolite concentration were evaluated using linear mixed‐effects modeling. The final model included intercept with no random effects and slope parameters for both centanafadine and the EB‐10601 metabolite concentrations. Random effects were estimated on the slopes. Final model parameters are presented in Table 2. The slope of the C‐QTc relationship was not significant for centanafadine (−0.001 ms per ng/mL) or for the EB‐10601 metabolite (−0.0003 ms per ng/mL). The model was evaluated using plots of observed data and quantiles overlaid with linear mixed‐effects model predicted ΔΔQTcF (90% CI) for centanafadine and the EB‐10601 metabolite (Figure 4).

**Table 2:** Model Parameters for Linear Mixed‐Effects Model Between ΔΔQTcF Versus Centanafadine and EB‐10601 Metabolite Concentrations

Parameter	Estimate	*P* value	90% CI
Intercept (millisecond)	2.006	.04	0.369, 3.643
Centanafadine slope (ms/[ng/mL])	−0.00142	.11	−0.00286, 0.00002
EB‐10601 metabolite slope (ms/[ng/mL])	−0.00029	.63	−0.00131, 0.00072

ΔΔQTcF, placebo‐corrected change from baseline in QTcF (ie, QT interval using the Fridericia correction formula); CI, confidence interval; TDD, total daily dose.

**Figure 5:**
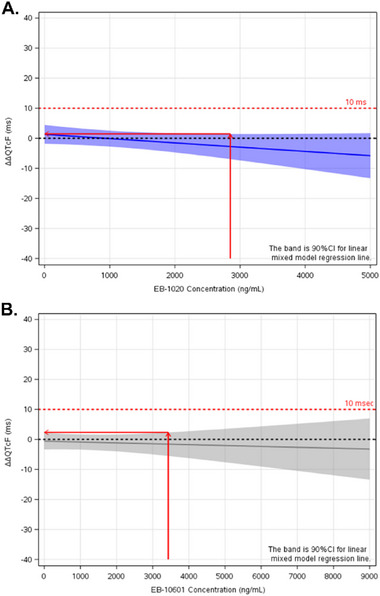
Predicted ΔΔQTcF (primary endpoint) based on linear mixed‐effects model for centanafadine (A) and the EB‐10601 metabolite (B), maximum plasma concentration (C_max_) values obtained from PK data set. Solid red lines indicate the ΔΔQTcF at the geometric mean C_max_; ΔΔQTcF, placebo‐corrected change from baseline in QTcF (ie, QT interval corrected using the Fridericia correction formula); CI, confidence interval.

The predicted ΔΔQTcF (90% CI) of centanafadine and the EB‐10601 metabolite using linear mixed‐effects model is presented in Table 3. At the geometric mean C_max_ of centanafadine 800 mg TDD (2.85 µg/mL), the predicted ΔΔQTcF was −2.72 milliseconds; at the geometric mean C_max_ of the EB‐10601 metabolite (3.43 µg/mL), the predicted ΔΔQTcF was −1.59 milliseconds. The upper bound of the 2‐sided 90% CI of the predicted ΔΔQTcF at the geometric mean C_max_ for both centanafadine and the EB‐10601 metabolite excluded 10 milliseconds (Figure 5).

**Table 3:** Predicted ΔΔQTcF (90% CI) for Centanafadine and EB‐10601 Metabolite from a Linear Mixed‐Effects Model

Analyte	Geometric mean C_max_ (µg/mL)	Predicted ΔΔQTcF (millisecond)	90% CI (millisecond)
Centanafadine	2.85	−2.72	−6.92, 1.48
EB‐10601 metabolite	3.43	−1.59	−5.52, 2.35

ΔΔQTcF, placebo‐corrected change from baseline in QTcF (ie, QT interval using the Fridericia correction formula); CI, confidence interval; C_max_, maximum plasma concentration, TDD, total daily dose.

Assay sensitivity was assessed by the moxifloxacin concentration and ΔΔQTcF relationship using a linear mixed‐effects model (Figure S6). The slope (90% CI) of the C‐QTc relationship for moxifloxacin was statistically significant (*P *= .0004), with an estimated slope of 0.004 ms/[ng/mL]. The predicted ΔΔQTcF at the geometric mean C_max_ of moxifloxacin following administration of moxifloxacin 400 mg (1.96 µg/mL) was 11.75 milliseconds (Figure S7).

### Safety

All reported treatment‐emergent AEs (TEAEs) were mild or moderate in severity (Table S2); all TEAEs were resolved by the end of the trial. The most frequently reported TEAEs (≥10% incidence rate) for 800 mg TDD of centanafadine SR were nausea (7/29 participants [24.1%]), dizziness participants (7/29 [24.1%]), headache (7/29 participants [24.1%]), and decreased appetite (4/29 participants [13.8%]). Three participants discontinued the trial, but none were attributed to AEs. No deaths or other serious AEs occurred. No clinically meaningful changes from baseline over time were observed in serum chemistry, hematology, urinalysis, vital signs, or ECG parameters. C‐SSRS assessment revealed no suicidal ideation or behavior during the trial.

## Discussion

In this TQT trial, the cardiac repolarization effect of twice‐daily centanafadine SR at the supratherapeutic dose of 800 mg TDD was studied in healthy adults. Neither centanafadine nor its EB‐10601 metabolite had a clinically meaningful effect on the QTc interval or on any other ECG parameters. Observed results excluded an effect on ΔΔQTcF up to plasma concentrations of 2.85 µg/mL for centanafadine and up to a plasma concentration of 3.43 µg/mL for the EB‐10601 metabolite. The concentrations measured resulted from taking a supratherapeutic dose of centanafadine SR (800 mg TDD), which is twice the maximum dosage used in clinical trials.^7^ Despite this, the predicted ΔΔQTcF values from centanafadine and its metabolite were −2.72 and −1.59 milliseconds, respectively, with the upper bounds of the 2‐sided 90% CIs of the predicted ΔΔQTcF being well below 10 milliseconds for centanafadine and the EB‐10601 metabolite, ruling out a clinically relevant QT interval effect with centanafadine.^18^ The moxifloxacin assay sensitivity confirmed the trial results.

These findings are consistent with results from QT trials that have been conducted on selective norepinephrine reuptake transporter inhibitors, atomoxetine and viloxazine, approved for the treatment of ADHD in adults.^19^, ^20^ Reuptake transporter inhibitors, whether selective or with inhibitory activity at multiple transporters, generally do not affect QT at clinically relevant doses.^21^, ^22^ However, citalopram, a selective serotonin reuptake inhibitor, is a notable exception.^23^ In contrast, amphetamines that are approved for the treatment of ADHD in adults have been associated with QTc prolongation.^24^

Analysis of secondary endpoints showed that centanafadine had no clinically meaningful effect on ECG parameters. Centanafadine SR was well tolerated in healthy adults at the supratherapeutic TDD of 800 mg. No serious TEAEs were observed, and all reported TEAEs were resolved by the end of the trial.

### Limitations

Participants received only 1 day of supratherapeutic dosing of centanafadine SR and were monitored for only 24 hours postdose, therefore conclusions cannot be drawn regarding the long‐term effect of centanafadine on cardiac repolarization.

## Conclusion

In this TQT trial in healthy adults, a supratherapeutic 800‐mg total daily dose of centanafadine SR had no clinically meaningful effect on cardiac repolarization or ECG parameters and was generally safe and well tolerated.

## Conflicts of Interest

O.S.T., J. R.‐G., and S.E.S. are employees of Otsuka Pharmaceutical Development & Commercialization, Inc. X.W. was an employee of Otsuka Pharmaceutical Development & Commercialization, Inc. at the time of the study.

## Funding

This trial was sponsored by Otsuka Pharmaceutical Development & Commercialization, Inc. (Princeton, NJ, USA)

## Supporting information



Supporting Information

Supporting Information

## Data Availability

To submit inquiries related to Otsuka clinical research, or to request access to individual participant data (IPD) associated with any Otsuka clinical trial, please visit https://clinical‐trials.otsuka.com/. For all approved IPD access requests, Otsuka will share anonymized IPD on a remotely accessible data sharing platform.
