# Supplementary material for: A Randomized Thorough QT Trial Using Concentration‐QT Analysis to Evaluate the Effects of Centanafadine on Cardiac Repolarization
Source: Clin Pharmacol Drug Dev. 2025 May 27;14(8):621–30. doi: 10.1002/cpdd.1545 (PMC12314114; doi:10.1002/cpdd.1545)
Supplement: Supplementary file 1 — Supporting Information [file CPDD-14-621-s001.pdf]

A Randomized Thorough QT Trial Using Concentration–QT Analysis to Evaluate the Effects of Centanafadine on Cardiac Repolarization

Osman S. Turkoglu[1], Xiaofeng Wang[1], Jennifer Repella-Gordon[1], Susan E. Shoaf[1]

[1]*Otsuka Pharmaceutical Development & Commercialization, Inc., Rockville, MD, USA*

SUPPLEMENTARY MATERIAL

Additional Exclusion Criteria

Participants who took an investigational medicine within 30 days of screening, had a history of significant bleeding, difficulty in donating blood or blood donation within 30 days, used antibiotics within 30 days, or used prescription, over-the-counter, or herbal medications, or vitamin supplements within 14 days of the first dose of trial drugs were excluded. Additionally, participants were excluded if they used substances known to stimulate hepatic microsomal enzymes within 30 days prior to screening to end of the trial, used tobacco products or had daily exposure to second-hand smoke within 2 months prior to the screening visit, had urine or serum cotinine concentrations of >200 ng/mL or >20 ng/mL, respectively, at screening or check-in, had a history of serious mental disorders, lacked a permanent place of residence, or had a previous exposure to centanafadine. Females who were breastfeeding or had a positive pregnancy test were excluded. The consumption of alcohol and/or food and beverages containing caffeine or methylxanthines, or foods known to affect CYP1A2 (e.g., charbroiled or pan-fried meats and cruciferous vegetables) within 72 hours prior to dosing disqualified participants from

enrollment. A history of drug/alcohol abuse within 2 years of screening or positive drug/alcohol testing at screening or admission; current hepatitis virus or AIDS, carriers of hepatitis B surface antigen and/or anti-hepatitis C virus, or HIV antibodies; and any significant drug allergy or known or suspected hypersensitivity were also exclusionary.

Table S1. Baseline Demographics

Parameter	Participants (N=30)
Age, mean (SD), years	37.6 (14.5)
Sex, n (%)	
Male	17 (56.7)
Female	13 (43.3)
Race, n (%)	
White	26 (86.7)
Other	4 (13.3)
Ethnicity, n (%)	
Hispanic or Latino	10 (33.3)
Not Hispanic or Latino	20 (66.7)
Body weight, mean (SD), kg	76.7 (16.2)
BMI, mean (SD), kg/m^2	26.4 (3.4)

SD, standard deviation; BMI, body mass index.

Table S2. Incidence of TEAEs Occurring in ≥10% of Participants Treated With Centanafadine SR 800 mg TDD, Moxifloxacin 400 mg, or Placebo (Safety Population)

Preferred MedDRA System Organ Class Term, n (%)	Centanafadine SR 800 mg TDD (n=29)	Moxifloxacin 400 mg (n=30)	Placebo (n=30)
Nausea	7 (24.1)	3 (10.0)	0
Dizziness	7 (24.1)	1 (3.3)	1 (3.3)
Headache	7 (24.1)	3 (10.0)	2 (6.7)
Decreased appetite	4 (13.8)	0	0
Palpitations	2 (6.9)	1 (3.3)	1 (3.3)
Diarrhea	2 (6.9)	0	0
Paresthesia	2 (6.9)	1 (3.3)	0
Change in sustained attention	2 (6.9)	0	0
Insomnia	2 (6.9)	0	0

SR, sustained release; MedDRA, Medical Dictionary for Regulatory Activities; TDD, total daily dose; TEAE, treatment-emergent adverse event.

Figure S1. Trial Design

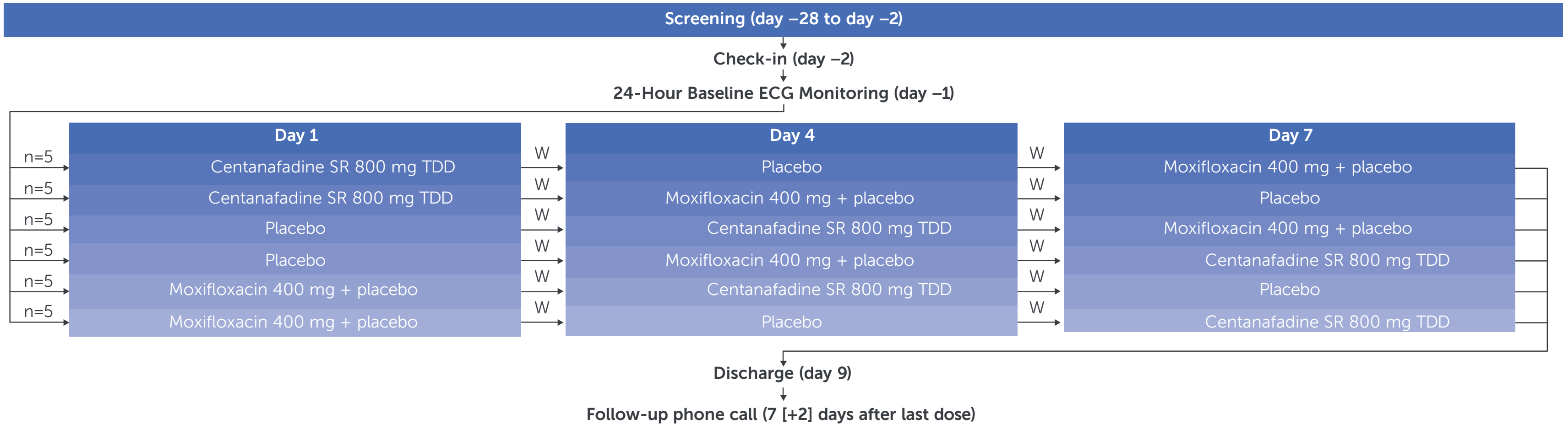

ECG, electrocardiography; SR, sustained release; TDD, total daily dose; W, washout period of ~72 hours between treatments.

Figure S2. LS Mean (90% CI) ΔΔHR (A) and ΔHR (B) Versus Time by Treatment

A.

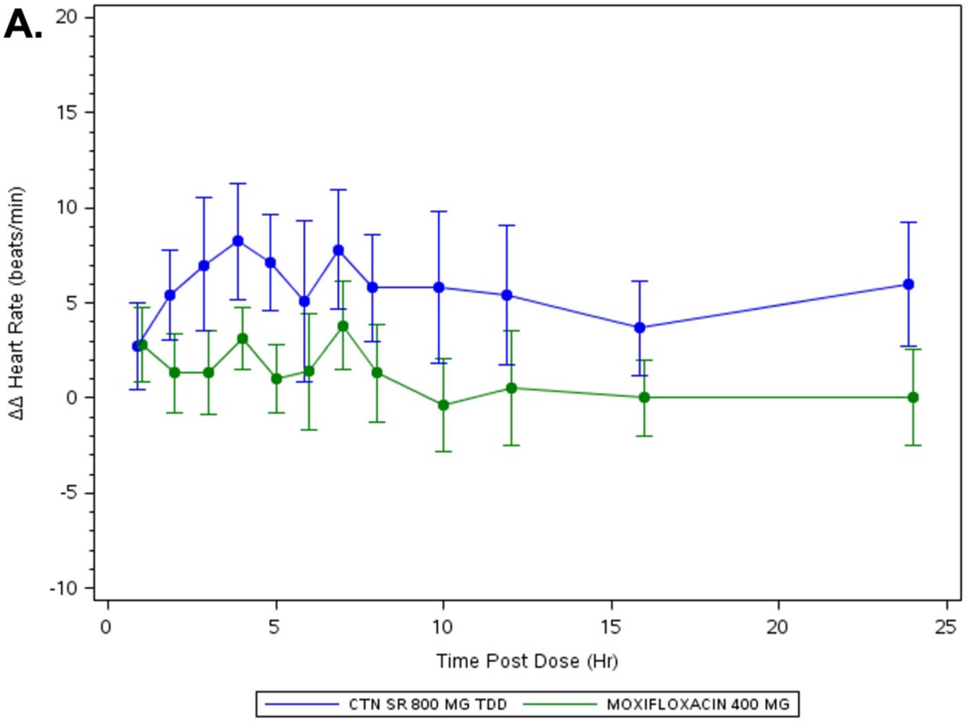

B.

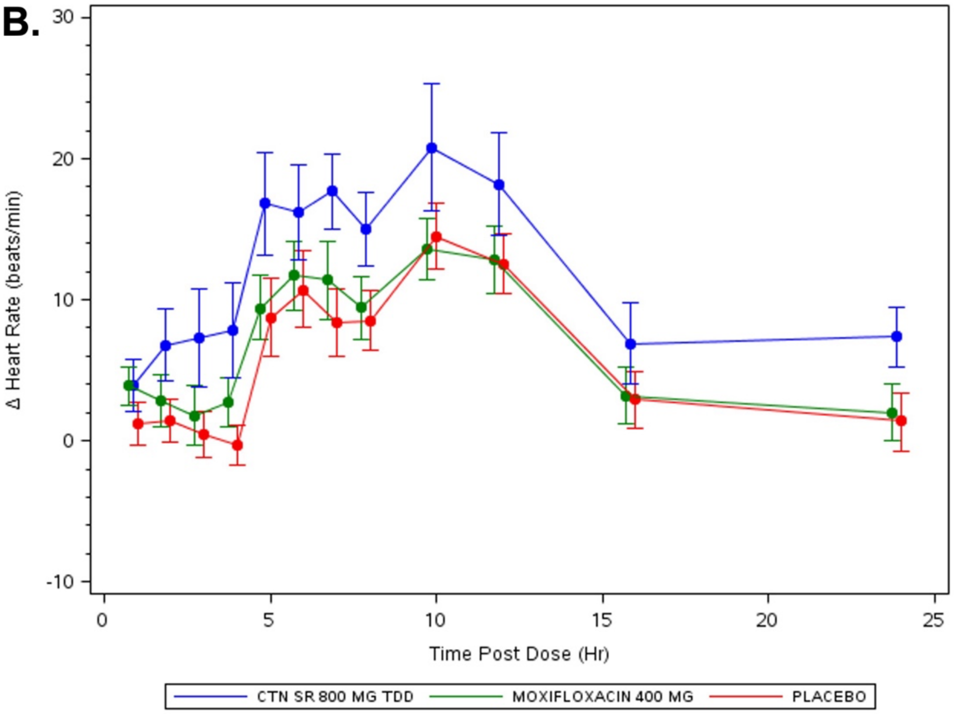

Error bars represent 90% CIs of the LS means. ΔΔHR, placebo-corrected change from baseline heart rate; ΔHR, change from baseline in heart rate; CI, confidence interval; CTN SR 800 MG TDD, centanafadine sustained release tablet 800 mg total daily dose; LS, least squares.

Figure S3. Scatter Plot of QTcF Versus RR Using Drug-Free Data (A) and Quantile Plot of Mean (90% CI) QTcF Versus RR (Median) Following Treatment Overlaid with Linear Mixed-Effect Regression Line and 95% CI (B)

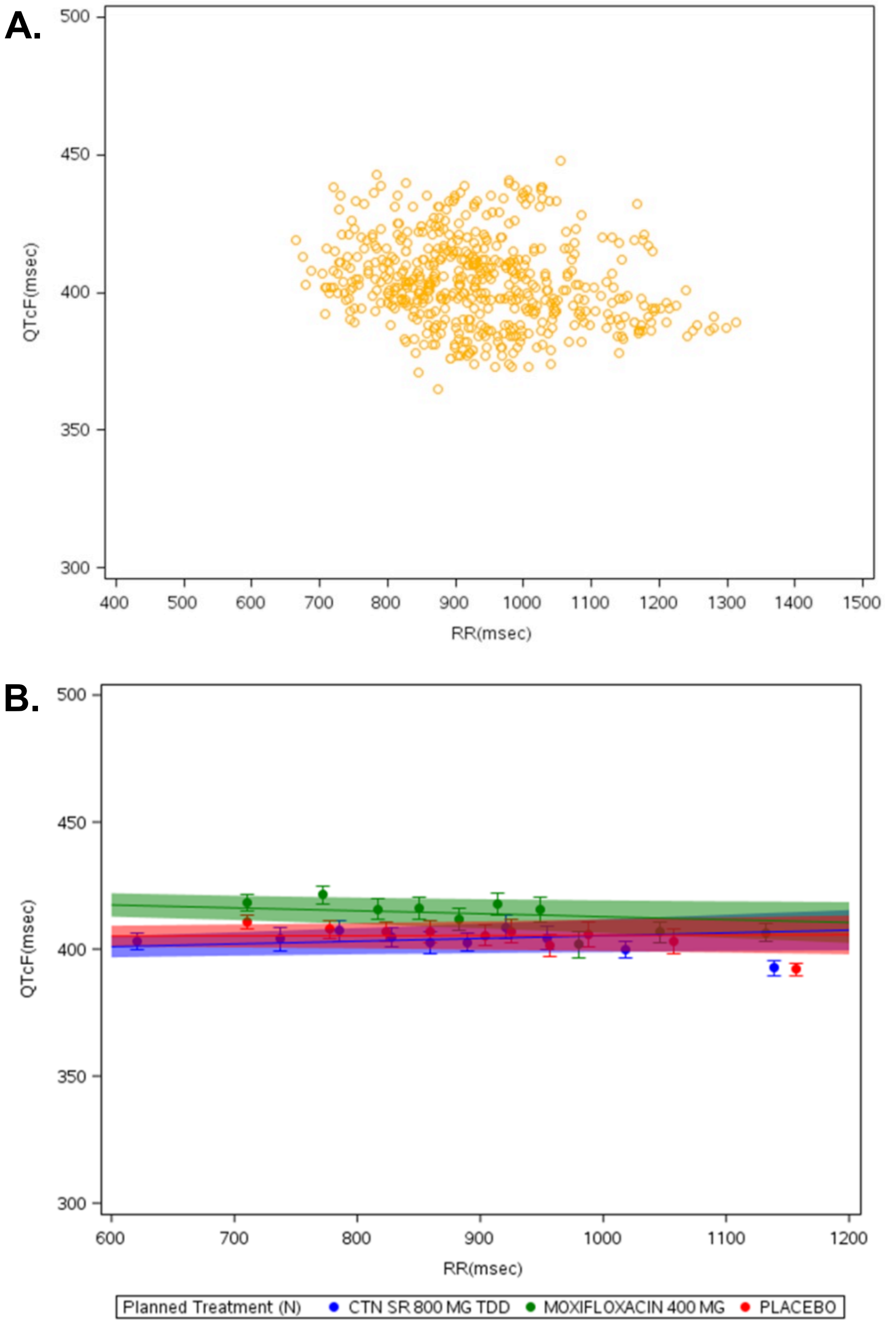

Drug free data includes values obtained on Day −1 and predose values collected at −0.75, −0.50, and −0.25 hours prior to dose on Day 1 of Period 1.
CI, confidence interval; CTN SR 800 MG TDD, centanafadine sustained release tablet 800 mg total daily dose; QTcF, QT interval corrected using Fridericia correction factor; RR, R wave to R wave interval.

Figure S4. Hysteresis Plots of Mean (90% CI) ΔΔQTcF Versus Concentration of Centanafadine (A) and EB-10601 (B)

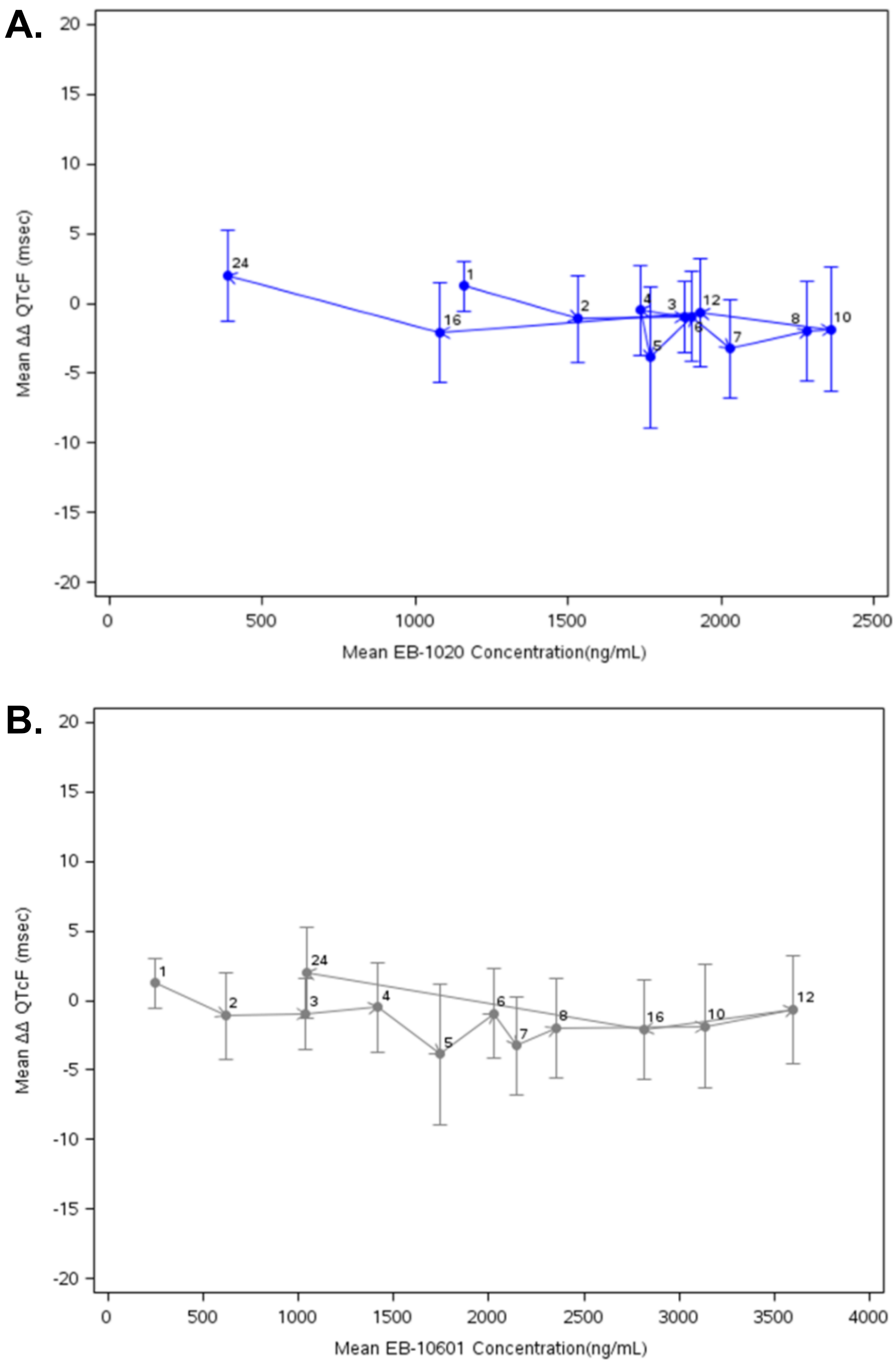

ΔΔQTcF, placebo-corrected change from baseline in QTcF (i.e., QT interval corrected using the Fridericia correction factor); CI, confidence interval.

Figure S5. Scatter Plots of Paired ΔΔQTcF for Centanafadine (A) and EB-10601 (B) Concentrations, Including Quantiles and Overlaid With LOESS and Linear Regression Lines Following 800 mg TDD of Centanafadine to Healthy Adults

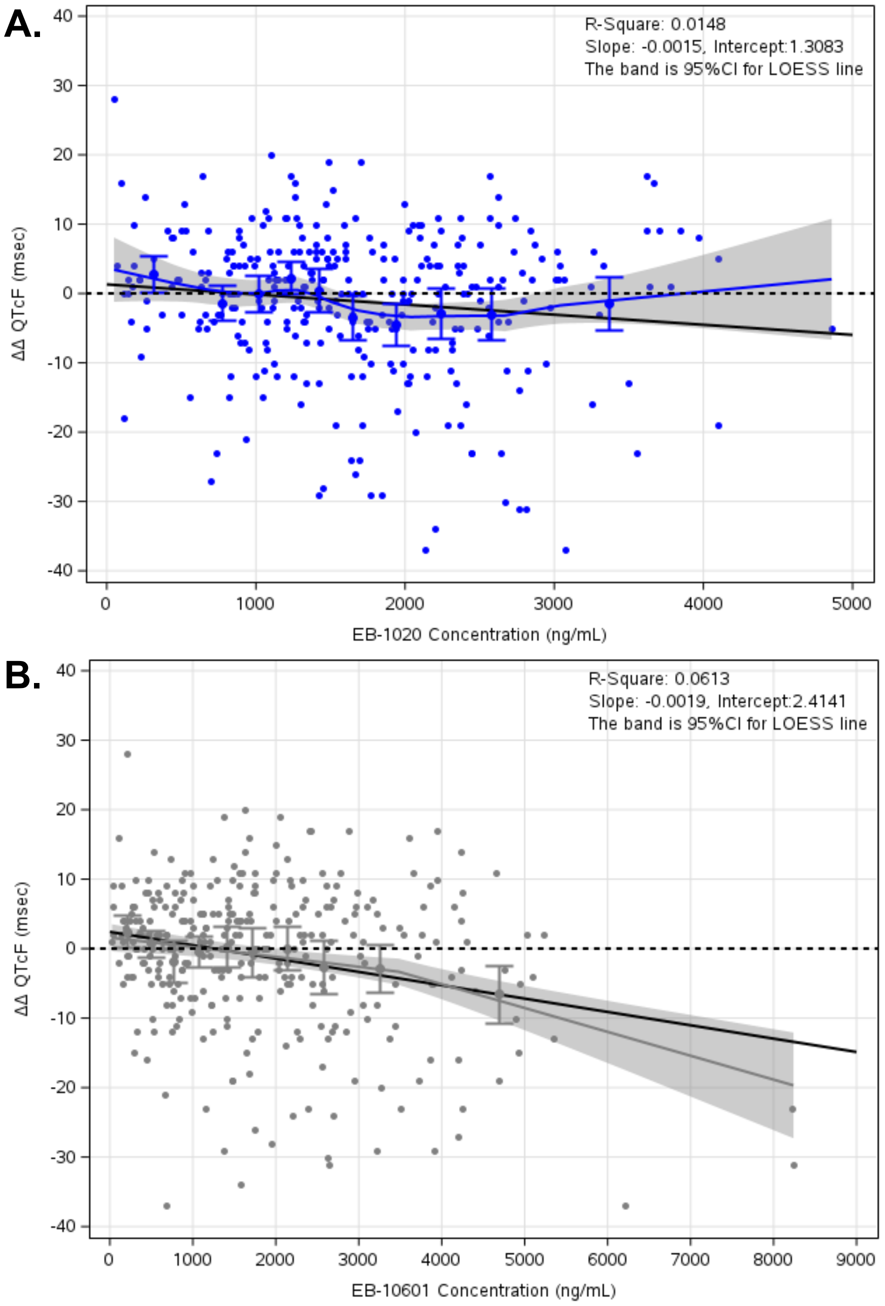

Solid circles denote individual data. Black line represents linear regression line. Quantiles denote mean concentration and mean ΔΔQTcF (90% CI). Blue or Gray line and band are LOESS regression line and 95%CI.

ΔΔQTcF, placebo-corrected change from baseline in QTcF (i.e., QT interval corrected using the Fridericia correction factor); CI, confidence interval; TDD, total daily dose.

Figure S6. Scatter Plot of Paired ΔΔQTcF and Moxifloxacin Concentrations, Including Quantiles and Linear Regression Line, Following Moxifloxacin 400 mg in Healthy Adults

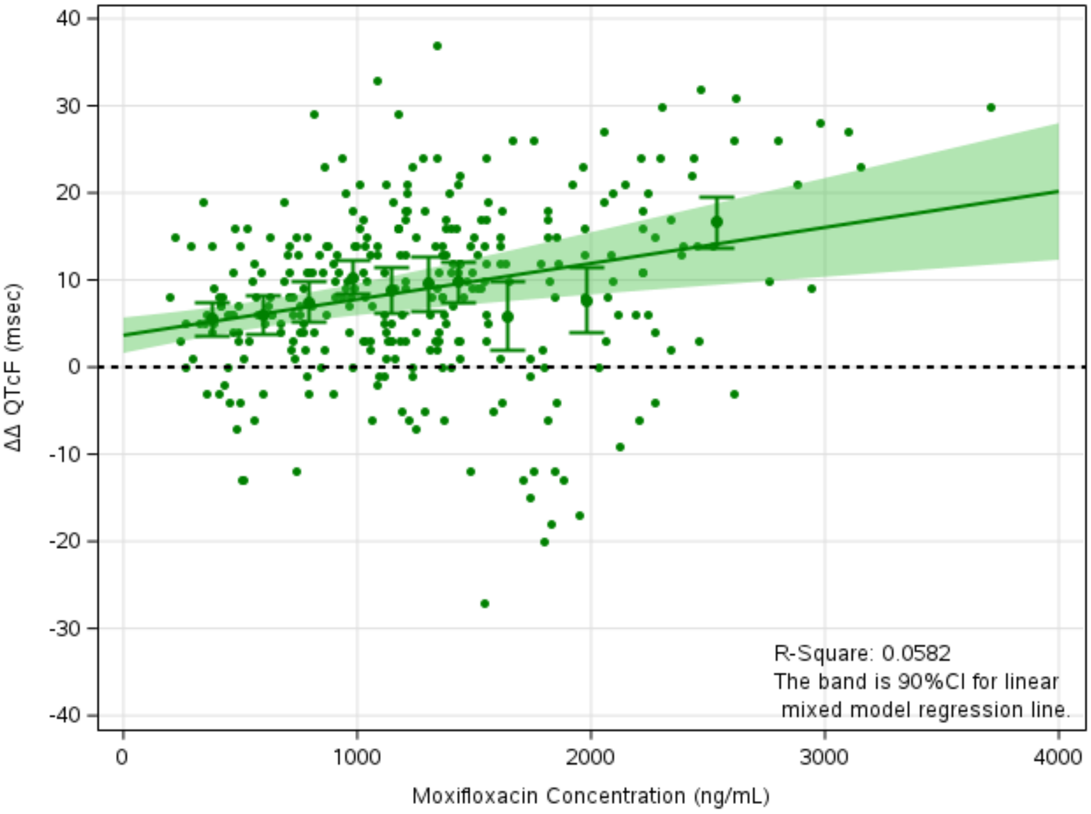

Solid circles denote individual data. Quantiles denote mean concentration and mean ΔΔQTcF (90% CI). ΔΔQTcF, placebo-corrected change from baseline in QTcF (i.e., QT interval corrected using the Fridericia correction factor); CI, confidence interval.

Figure S7. Predicted ΔΔQTcF Based on Linear Mixed-Effects Model for Moxifloxacin

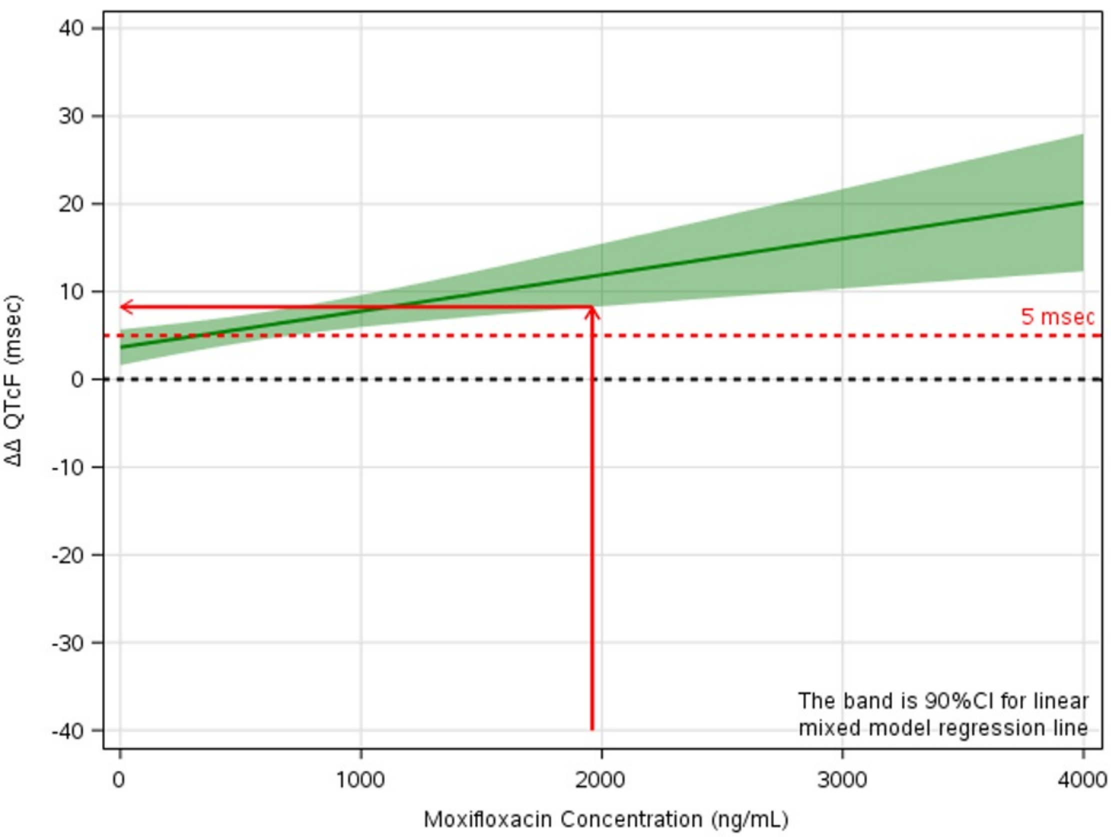

Red solid line shows predicted ΔΔQTcF at geometric mean C_{max} of moxifloxacin obtained from PK dataset. The predicted ΔΔQTcF at the geometric mean (90% CI) C_{max} of moxifloxacin following administration of moxifloxacin 400 mg (1.96 µg/mL) was 11.75 (8.25, 15.24) msec.

ΔΔQTcF, placebo-corrected change from baseline in QTcF (i.e., QT interval corrected using the Fridericia correction factor); CI, confidence interval.
